# Current Status of the Diagnosis of *Brugia* spp. Infections

**DOI:** 10.3390/pathogens13090714

**Published:** 2024-08-23

**Authors:** Christopher C. Evans, Nils Pilotte, Andrew R. Moorhead

**Affiliations:** 1 Department of Infectious Diseases, College of Veterinary Medicine, University of Georgia, Athens, GA 30602, USA; 2 Department of Biological Sciences, Quinnipiac University, Hamden, CT 06518, USA; nils.pilotte@quinnipiac.edu

**Keywords:** diagnostics, filarial nematodes, lymphatic filariasis, *Brugia*, zoonoses

## Abstract

Filarial nematodes of the genus *Brugia* include parasites that are significant to both human and veterinary medicine. Accurate diagnosis is essential for managing infections by these parasites and supporting elimination programs. Traditional diagnostic methods, such as microscopy and serology, remain vital, especially in resource-limited settings. However, advancements in molecular diagnostics, including nucleic acid amplification tests, offer enhanced sensitivity and specificity. These techniques are becoming increasingly field-friendly, expanding their applications in diagnostics. By refining existing methods, developing novel biomarkers, and understanding the zoonotic potential of various *Brugia* species, it is possible to improve control measures and better support elimination efforts.

## 1. Introduction

The filarial nematodes (superfamily Filarioidea) encompass a diverse group of parasites that are significant to both human and veterinary health. Their life cycles involve transmission through blood-feeding arthropods, with the adult stage often persisting for extensive periods in the definitive host. Females release vermiform embryos called microfilariae that migrate to the peripheral blood stream or skin, depending on the species, wherein these microfilariae may be taken up by a suitable vector. Filarial parasite infections are often asymptomatic and go undetected but may in some cases become life-threatening.

Within the diversity of filarial nematodes, the genus *Brugia* [1] stands out for its medical importance, zoonotic potential, widespread distribution, and utility in research settings. Notable among the ten described species to date are *B. malayi* and *B. timori*, well-recognized causative agents of lymphatic filariasis in humans, one of the most debilitating of the neglected tropical diseases, affecting millions worldwide [2]. The impact of worms within this genus extends beyond human health, parasitizing domestic and wild animals while posing zoonotic threats in endemic regions [3].

The complex life cycles of filarial worms and their localization within the host results in unique diagnostic challenges, and a variety of tests have been developed as an understanding of their biology and the availability of molecular techniques has advanced (see Section 5). Given the diverse clinical manifestations and their variable severity, accurate diagnosis by identification of the parasite is crucial to managing filarial infections [4]. This not only allows targeted antifilarial treatment in individuals, but also supports surveillance efforts to assess infection prevalence and monitor progress toward elimination goals. Additionally, accurate diagnostic techniques facilitate research into epidemiology, transmission dynamics, and treatment strategies for these parasites.

This review aims to comprehensively detail the existing diagnostic techniques available for detection of *Brugia* spp. infection with an emphasis on accuracy and utility. Techniques in both human and veterinary medicine will be addressed, including methods for distinguishing similar species where overlap may exist. We will also highlight recent advancements in filarial diagnostics and their potential for improving detection and management strategies.

## 2. Epidemiology and Distribution of *Brugia* spp. Infections

The global distribution and burden of filarial nematodes are significant public health concerns. An estimated 51 million individuals are infected with species that cause lymphatic filariasis [5] with about 880 million people in 44 countries at risk [2]. While *Wuchereria bancrofti* is the most common causative agent of the disease, approximately 10% of cases are due to infection with *B. malayi* or *B. timori* [2]. The impact of *Brugia* spp. parasites on animal health is incompletely understood, and the precise role of domestic and sylvatic hosts as reservoirs for human filariases and sources of zoonotic infection is still emerging. Filarial nematodes thrive in tropical and subtropical climates where suitable mosquito vectors are abundant. Efforts to map the global distribution of *Brugia* spp. infection have identified endemic regions in South and Southeast Asia as well as parts of Africa and the Americas [5,6], however, gaps in surveillance and reporting may underestimate the true range of these parasites. Here, effective diagnostics are essential.

The species of greatest concern to human health, *B. malayi*, is endemic to India and Southeast Asia [2,7]. Domestic cats also serve as competent hosts, with prevalence as high as 20% reported in endemic populations [8,9]. Primates, wild felids, civets, and pangolins are also known hosts with the potential to serve as reservoirs [10]. The range of *B. timori* is restricted to the eastern islands of the lesser Sunda archipelago, Indonesia and Timor-Leste; no known animal reservoir exists for this species [11].

*Brugia pahangi* is a natural parasite of felids, occurring in India and Southeast Asia. The reported prevalence in domestic cats ranges from 11 to 25% [8,12,13,14]. Other hosts include primates, wild felids, and civets, which may serve as sylvatic reservoirs [15]. *Brugia ceylonensis* is a parasite of dogs with implicated zoonotic potential [16]. This species has only been identified in Sri Lanka, with one survey reporting its prevalence at 7% in domestic dogs [17]. Also endemic to Sri Lanka, *B. buckleyi* is a parasite only reported endemically in the Indian hare (*Lepus nigricollis*) [18,19], though one report suggests adults of this parasite were recovered from an Asian small-clawed otter (*Aonyx cinereus*) originating from Malaysia [20]. *Brugia tupaiae* occurs in Southeast Asia, largely overlapping the range of its definitive host, treeshrews of the genus *Tupaia* [21,22,23].

The only species endemic to Africa, *Brugia patei*, is found on Pate Island, Kenya, and nearby mainland coastal regions. It has been recovered from domestic dogs and cats, as well as genets (*Genetta tigrina*) and galagos (*Galago crassicaudatus*) [24,25]. Infection rates on Pate Island are reported to be higher for cats (72%) and dogs (25%) [26] than on nearby coastal regions ranging from Somalia to Tanzania (16 and 6%, respectively) [25]. More recently, worms genetically similar to *B. patei* have been recovered from dogs in Chad [27].

Two described species of *Brugia* are endemic to North America. *Brugia beaveri* has been identified in the United States from Louisiana to Florida and is a natural parasite of raccoons (*Procyon lotor*), with one study reporting a prevalence of 70% [28]. It has also been identified in the bobcat (*Lynx rufus*) and mink (*Neogale vison*) and has been demonstrated to fully develop in domestic cats [28,29]. *Brugia lepori* (Syn. *B. leporis*) is endemic to Louisiana and parasitizes cottontail rabbits (*Sylvilagus* spp.) with a prevalence of 71% [30]. The true range of these species remains unknown and warrants further investigation. Human cases of *Brugia* spp. infection have been reported across the United States in individuals with no history of travel to endemic regions [6,31,32]. As such, *B. beaveri*, *B. lepori*, and perhaps other as yet undescribed species may be regarded as potential zoonotic agents.

A single species, *B. guyanensis*, is endemic to South America. It has been identified in Guyana in the South American coati (*Nasua nasua*) and greater grison (*Galictis vittata*) [33,34]. A single report of human infection describes parasites consistent with *B. guyanensis* recovered from the patient following a trip to the Amazon basin of Peru, potentially indicating not only an expanded range for the species, but also its zoonotic potential [35]. Hosts and geographic ranges for all described *Brugia* spp. are presented in Table 1.

Several factors influence the transmission of filarial parasites, thus affecting their distribution and persistence within human and animal populations. The distribution and abundance of competent vectors, which include mosquitoes from the genera *Aedes*, *Anopheles*, *Culex*, and *Mansonia*, are crucial to the transmission of *Brugia* spp. parasites [40]. The vectorial capacity of each mosquito species, a measure of its ability to transmit parasites, is influenced by factors such as longevity, biting frequency, and vector competence [41,42,43]. High vectorial capacity enhances the efficiency of parasite transmission and contributes to the sustained presence of filariasis in endemic areas. Vector abundance also influences geographic distribution, which itself is governed by environmental factors such as temperature, humidity, and rainfall. This generally limits *Brugia* spp. distribution to the tropics and subtropics [7,41]. Urbanization has affected mosquito breeding habits and host-vector interactions. Urban areas with inadequate sanitation and drainage systems may provide favorable conditions for mosquito breeding and increased transmission potential [44]. The availability of suitable mammalian hosts is always crucial to the maintenance of filarial populations, which will be affected by population density and the implementation of transmission control measures, including mass drug administration.

## 3. Life Cycle and Pathogenesis of *Brugia* spp.

The mosquito vector takes up microfilariae in the peripheral circulation during a bloodmeal, which penetrate the midgut wall and shed their sheaths [45]. Over the course of approximately 10 days, microfilariae subsequently migrate to the flight muscles, molting twice and developing to infective third-stage larvae localized in the mouthparts of the mosquito [46]. Transmission to the definitive host occurs during blood feeding, when infective larvae emerge from the mouthparts and are deposited onto the skin of the host, migrating into the fresh bite wound and reaching the lymphatics in as little as 3 days [47]. In *B. malayi*, the best-studied member of the genus, the molt to the fourth larval stage occurs at 8–10 days postinfection, followed by the final molt to the adult stage 35–40 days postinfection [48]. In the cat, *B. malayi* microfilariae are first observed between 70 and 147 days postinfection [49,50]. In competent hosts, adults remain in the lymphatics, releasing microfilariae that return to the peripheral blood where they may be taken up by the mosquito vector and continue the life cycle [51]. It is possible to visualize the adult stage of *Wuchereria bancrofti* by ultrasonography but not *B. malayi* [52]. In cases of zoonotic filariasis, in which the human host may or may not be permissive to infection, mature and immature worms have been recovered from biopsied lymph nodes and, rarely, extra lymphatic tissue [6,31,53,54,55].

The presence of adult worms in the lymphatics may cause pathology by occluding vessels, and in humans, clinical manifestations may take multiple forms. Acute filariasis is episodic in nature and may include lymphadenitis, lymphangitis, and subsequent lymphedema. Chronic pathology may develop years after initial infection, in which lymphatic tissue damage and subsequent bacterial and fungal infections promote the development of elephantiasis [56,57]. Rarely, tropical pulmonary eosinophilia may develop, characterized by asthma-like symptoms [58]. Filariasis presents across a spectrum with two distinct extremes. The majority of cases are asymptomatic yet microfilaremic, whereas individuals with chronic pathology are more commonly amicrofilaremic [4,59,60,61]. This has been explained as a reflection of the parasite succeeding or failing, respectively, to effectively modulate the immune response of its host [62,63]. There also exist asymptomatic ‘latent’ infections, in which adult parasites are present, but not circulating microfilariae [60,64].

Very little has been reported on the clinical manifestations of *Brugia* spp. infection in animals. Lymphedema has been described in cats and ferrets infected with *B. malayi* [65,66]. Another study reports no thickening of lymph vessels nor inflammation associated with an unidentified *Brugia* sp. in a domestic cat from California [55]. A study on *B. malayi* in dogs reports no clinical signs [38]. The pathogenesis of filarial infection in wild hosts remains understudied.

## 4. Classical Diagnostic Methods for *Brugia* spp. Infections

The diagnostic techniques applied to filarial parasites today were developed as a result of extensive research into their life cycles, geographic ranges, and molecular characteristics. In endemic regions, classical diagnostic methods have historically been essential to the identification and monitoring of filariasis and remain relevant today. These techniques are used in resource-limited settings where access to more sophisticated equipment and reagents may be unavailable. What follows is a review of the principles, applications, and limitations of classical microscopic and serological methods for diagnosis of *Brugia* spp. infection.

### 4.1. Microscopy

The simplest method for detecting microfilaremic infection, still commonly employed in clinical settings, is the direct blood smear technique. This straightforward approach involves placing a drop of anticoagulated blood onto a glass slide, coverslipping, and examining under a microscope [67]. While the forms of the microfilariae are challenging to visualize directly in such preparations, their motility can be more easily observed as they agitate the surrounding erythrocytes. While direct smears can be useful when microfilaria levels are high, they may fail to detect parasites at lower concentrations, and this limitation is exacerbated when microfilariae exhibit periodicity. This is a non-specific method of detection and cannot be used to effectively distinguish one *Brugia* sp. from another.

The phenomenon of microfilarial periodicity is characterized by the circadian rise and fall of microfilariae in the peripheral circulation, sometimes dropping so low as to be undetectable. Peak microfilaremia is thought to coincide with peak feeding times of locally significant mosquito vectors [68,69]. *Brugia malayi* is a nocturnally periodic species with two strains having been described, periodic and subperiodic, characterized by the level of microfilarial depletion during daylight hours. It appears that only the latter strain naturally occurs in cats [70]. Nocturnal periodicity has also been described in *B. timori* infection [71]. *Brugia pahangi* is described as subperiodic, and consequently, blood samples for diagnosis can be taken at any time of day and be expected to yield microfilariae [72,73]. Diurnal periodicity has been observed in *B. tupaiae* infection of treeshrews, while no periodicity has been observed in *B. beaveri* infection of raccoons [28]. It has been proposed that pronounced nocturnal periodicity is a characteristic infection in primate hosts but not other animals [3]. Microfilarial periodicity and its varied manifestations led to the development of concentration techniques, which enable parasite detection at any time of day.

The Knott test was initially developed to detect the nocturnally periodic microfilariae of *W. bancrofti* [74]. In this procedure, 1 mL of anticoagulated venous blood is mixed with 10 mL of 2% formalin solution. This mixture serves the dual purpose of lysing erythrocytes to enhance parasite visibility and preserves the sample for subsequent examination. After centrifugation, the supernatant is discarded, and the pellet is stained with methylene blue or a similar dye. The stained blood sample can then be microscopically examined, either with a coverslip or after air drying. Known as the ‘modified Knott test’ in veterinary medicine, Knott’s concentration technique is recommended for detecting blood-dwelling microfilariae due to its simplicity, affordability, and standardization. Microfilariae observed through this method can be measured and compared against established diagnostic criteria (such as length and width) for species identification.

However, distinguishing microfilariae of closely related species based solely on morphology can be challenging, and in such instances, the localization of acid phosphatase activity may prove useful. This histochemical technique has been employed to differentiate similar species for taxonomic classification and subsequent diagnostics [75,76]. In recent years, there has been a resurgence in its application for assessing suspected *B. malayi* infections in dogs, serving as a supplementary method alongside morphological and molecular analyses [11,12,13].

More simplified concentration techniques have also been developed, though they are less commonly utilized. One such method involves filtration of blood through a nucleopore filter, from which microfilariae can be washed and observed [77]. Additionally, the centrifugation of blood in microhematocrit tubes and examination of the buffy coat has been described. The microfilariae that concentrate in this layer may be observed directly or stained for visualization of terminal nuclei, which can distinguish similar species [78,79,80].

Methods such as the thick blood smear allow for the calculation of microfilaria concentration in venous blood by staining and examining known volumes (typically 20 µL). As a less common alternative, the volume of the pellet obtained from a Knott test can be measured and examined. Determining an accurate microfilaria concentration is mainly useful for research purposes, but there are specific clinical scenarios in which it may also prove valuable. High levels of microfilariae increase the risk of anaphylactic reactions against parasite antigens released during treatment and may alter treatment decisions. However, the examination for microfilariae primarily serves as a qualitative diagnostic parameter that also aids in species identification and assessing transmission potential. At the time of this writing, the World Health Organization (WHO) recommends thick blood smears of finger-prick blood as the diagnostic technique of choice for detecting *Brugia* spp. in humans [67].

Several characteristics have been described that help differentiate *Brugia* spp. from other filarioids, and one species of *Brugia* from another where their distributions overlap. The microfilariae of all *Wuchereria* spp. and *Brugia* spp. can be found in the blood and are surrounded by a characteristic hyaline sheath that is nearly transparent unless stained. While this can guide identification, it should be noted that *Loa loa* and *Litomosoides* spp. microfilariae are also sheathed. Distinguishing *B. malayi* from other species with less zoonotic potential is important, and reports on suspected canine infections have included differential techniques for *B. pahangi* and *B. ceylonensis*, which are co-endemic and more likely to be seen in dogs [12,81,82]. In feline infections, it has typically been observed that *B. malayi* microfilaremia is lower than *B. pahangi*, though it is not certain how generalizable this finding may be [83]. Additionally, anatomical features like the innenkorper (central viscus) can be visualized by staining and this structure is shorter in *B. malayi* than *B. pahangi* [84]. *Brugia* spp. may also be distinguished by the anatomical localization of acid phosphatase activity. This histochemical technique reveals two foci of staining (excretory and anal pores) in *B. malayi* microfilariae, diffuse staining throughout *B. pahangi*, and staining in the cephalic vesicle, excretory pore, and tail of *B. patei* [76,85]. Diagnostic acid phosphatase staining has not been described in all *Brugia* species and has primarily seen application in assessing zoonotic threats posed by *B. malayi* [12,81,82]. Numerous morphological features have been used to describe and differentiate *Brugia* spp. parasites in both microfilarial and adult stages, and while a comprehensive treatment of all these features is beyond the scope of this review, a selection of diagnostic parameters have been summarized in Table 2.

### 4.2. Serology

Serological techniques offer a high-sensitivity alternative to microscopy-based diagnostics by detecting circulating parasite antigen or host antibodies to the parasite. Such techniques do not rely on the presence of circulating microfilariae, nor are they subject to the effects of microfilarial periodicity [89]. Serology-based diagnostic tools can provide rapid results with relatively little need for clinical infrastructure, and while microscopy techniques are inexpensive and highly specific, their sensitivity is relatively low. Antigen and antibody presence and concentration can potentially be used as a measure of transmission intensity and continued exposure to infection, regardless of microfilaremia status, and as such, these methods are very useful for monitoring progress in elimination efforts [90,91,92].

A test for circulating filarial antigen exists for *W. bancrofti*, but no such equivalent has been used for detecting *Brugia* spp. infections [93]. Instead, serology relies on detection of host antibodies to filarial antigen. It has been observed that individuals living in filariasis-endemic regions present with elevated IgG4 antibodies to known parasite antigens, even when microfilariae and the antigens themselves are not detectable [94]. As such, exposure to *Brugia* spp. parasites in humans can be detected by exploiting this specific IgG4 serology with the aid of recombinant parasite antigens via multiple methodologies, detailed in the following paragraphs.

Immunochromatographic tests are used in the detection of parasites causing lymphatic filariasis in humans. The commercially available Brugia Rapid test (Reszon Diagnostics International, Subang Java, Selangor, Malaysia) was developed with a focus on *B. malayi* infection, detecting antibodies to the recombinant BmR1 antigen [95]. Evaluations of the test have reported sensitivity up to 100% and specificity again *O. volvulus* and *L. loa* up to 98.8% and 100%, respectively, though these species are not co-endemic with *B. malayi* and not likely to produce false positives [90,96,97]. The Brugia Rapid test is also sensitive to *B. timori* infection [98]. The PanLF Rapid test (Reszon Diagnostics International, Subang Java, Selangor, Malaysia) detects antibodies to the recombinant antigen BmSXP in addition to BmR1, allowing detection of both brugian and bancroftian filariasis. Sensitivity up to 100% has been reported for *B. malayi*, and again, some cross-reactivity is observed with *O. volvulus* and *L. loa*, with a specificity of 99% [99].

In research settings, ELISA has been used to demonstrate the sensitivity of *Brugia* spp. antibody detection [100,101]. The Filariasis Cellabs Enzyme Linked Immunosorbent Assay (CELISA; Cellabs, Brookvale, New South Wales, Australia) was developed and released as a commercially available test. It detects antibodies to the recombinant filarial antigen Bm14, which are indicators of both *W. bancrofti* and *B. malayi* infection, with 98% and 91% sensitivity in microfilaremic cases, respectively [102]. The CELISA assay uses a 96-well plate format, in which samples (serum, plasma, or blood spot eluate) are added to wells coated with recombinant antigen and incubated with peroxidase-conjugated antibody to human IgG4, to which a chromogenic substrate is added allowing visual or spectrophotometric readings. Some cross-reactivity to *O. volvulus* and *L. loa*, as well as non-filarial nematodes of the genera *Ascaris* and *Strongyloides* has been reported [90,102].

At the time of this writing, the WHO recommends the Brugia Rapid test as the serological test of choice for detecting *Brugia* spp. in humans [67]. No commercially available tests are validated for detecting *Brugia* spp. infections in animals. In cat infections, however, experimental use of indirect immunofluorescence has been described for *B. malayi*, while ELISA and immunoprecipitation techniques have been described for *B. pahangi* infection [103,104,105].

## 5. Nucleic Acid Amplification Tests for the Detection of *Brugia* spp. Infections

Providing an alternative to microscopy- and serology-based diagnostic methods, the use of nucleic acid amplification tests (NAATs) has become increasingly common for detection of filarial, and more broadly, parasitic infection [106]. Relying on the enzymatic amplification of target nucleic acid sequences that (ideally) enable sensitive and specific detection of a pathogen, NAATs, when properly designed, are commonly held to represent the most accurate diagnostic tests available. However, the widespread use of NAATs has historically been hampered by increased instrumentation requirements, cost concerns, training needs, and reliable cold chain availability [107]. With the development of more field-friendly approaches to nucleic acid detection and the advent of increasingly stable reagents, the perception of these tests is beginning to change, and their potential as broadly available and implementable assays is increasing [108].

### 5.1. Laboratory-Based Nucleic Acid Amplification Tests

To date, molecular diagnostic tests designed for the detection of *Brugia* spp. have been limited almost exclusively to detection of the human-infecting pathogens *B. malayi* and *B. timori* and the occasionally zoonotic pathogen *B. pahangi*. Coupling conventional polymerase chain reaction (PCR) techniques with agarose gel electrophoresis, the first *Brugia* spp. detecting molecular diagnostic test was developed in the mid-1990s for the detection of *B. malayi* in human blood [109]. The development of a PCR-ELISA assay for the detection of *B. malayi* soon followed, and its utility for detection of pathogen from both human blood samples and mosquito samples was explored [110,111,112]. In the mid-2000s, a real-time PCR-based assay for the detection of *Brugia* DNA in human blood was first described [113], with detection in vector mosquitoes soon following [114]. This assay became (and remains) the benchmark for NAAT-based detection of human *Brugia* infection. An RNA-targeting NAAT has also been described for the detection of *B. malayi*, with the goal of facilitating stage-specific detection of L3 parasites in mosquitoes [115]. This assay differentiates between “infected” and “infective” vectors, a potentially important distinction with implications for transmission potential, in turn impacting programmatic decision making. However, to date, the use of this assay for operational research or programmatic purposes has not occurred. Limited efforts to apply NAAT-based detection methods to the human-infecting parasite *B. timori* have relied largely on assays designed for *B. malayi* detection. With a shared DNA target sequence (the Hha I repeat), such assays are capable of detecting *B. timori* in addition to *B. malayi* [116], although additional testing is required to differentiate the two parasites at the species level [11]. This target is also partially conserved across other *Brugia* species, complicating efforts to differentiate the human-infecting pathogen from parasites with non-human hosts when testing mosquitoes and/or samples derived from animal reservoirs [117,118]. Such conservation has led to efforts to distinguish *B. malayi* and *B. timori* from other *Brugia* spp., and assays capable of differentiating *B. pahangi* from the human-infecting parasites have been described [119,120].

### 5.2. Field-Friendly Nucleic Acid Amplification Tests

With equipment and infrastructure needs presenting a challenge for implementation of laboratory-based NAATs in many settings, efforts aimed at the development of field-friendly diagnostics facilitating use at the point of sample collection have vastly expanded in recent years [108]. To date, published efforts to develop PCR-free techniques (thus eliminating the need for temperature cycling and the associated instrumentation) have focused on the detection of human-infecting species utilized loop-mediated isothermal amplification (LAMP) strategies coupled with colorimetric detection [121,122]. However, technological advancements facilitating the development of field-friendly PCR and real-time PCR instrumentation has led to a partial shift in the design strategy for point-of-collection-based tests. While efforts to adapt new amplification strategies continue to occur, adaptation of existing strategies (PCR and real-time PCR) to the field have also expanded [123]. Such efforts aim to couple field-friendly DNA extraction techniques with miniaturized PCR equipment and lateral flow detection. At the time of publication, one such assay, facilitating the field-friendly detection of *B. malayi* from mosquitoes has been described [124] and additional development efforts are underway.

### 5.3. Future Perspectives on Nucleic Acid Amplification Tests

Recent analyses have suggested that animal reservoirs of human-infecting *Brugia* spp. may complicate transmission interruption in regions with zoonotic infection [125]. Given these likely challenges, fully understanding the prevalences in both human and animal hosts, as well as the transmission dynamics underpinning these interactions will be critical to shaping and monitoring intervention efforts. The development of diagnostic assays capable of addressing such uncertainties will be critical to future efforts. To facilitate their widespread use, such diagnostics will need to be field-friendly and cost-effective. Additionally, they will need to differentiate *Brugia* parasites to the species level, which will be critical both for assessing whether animal infections represent possible zoonotic reservoirs, and for mosquito monitoring efforts where mixed populations of human-infecting and strictly animal-infecting pathogens could complicate an understanding of prevalence. While currently utilized NAATs for *Brugia* spp. diagnostics rely almost exclusively on amplification of the Hha I repetitive element, species level differentiation may require the identification of new target sequences capable of distinguishing between species.

## 6. Novel Biomarkers and Targets

As described by the WHO lymphatic filariasis diagnostic technical advisory subgroup, target product profiles (TPPs) for the development of improved diagnostics include the identification of novel biomarkers for human-infecting *Brugia* spp. [126]. For intervention “stopping” decisions, such biomarkers must be capable of identifying live worms that are capable of reproduction from those that are dead or permanently sterilized. As such, this biomarker could take the form of an antigen produced only by reproduction-capable worms, or of an RNA species underlying such an antigen [126]. In contrast, for post-intervention surveillance efforts, antibodies specific for early exposure or biomarkers (antigen or RNA) indicative of pre-patent infection would be required to differentiate new infections from those that are long established [126]. Given the WHO-endorsed backing of such TPPs and the reliance upon WHO guidelines for the steering of programmatic intervention and monitoring efforts, the identification of such biomarkers should be a priority.

## 7. Conclusions

The diagnosis of *Brugia* spp. infections requires a multifaceted approach due to the complexity of the parasites’ life cycles and the variability in clinical presentations. Classical diagnostic methods remain fundamental, especially in settings with limited resources. However, advancements in molecular methods have provided more sensitive and specific diagnostic options, with recent developments making these tests increasingly field friendly.

Future research should focus in part on refining existing diagnostic tools to enhance accessibility and cost-effectiveness, but even more important is the identification of novel biomarkers to enable diagnosis of infections of greatest relevance. Additionally, the development of species-specific assays could allow more accurate epidemiological studies and intervention strategies. Understanding the zoonotic potential of the various *Brugia* spp. and the roles played by animal reservoirs in transmission is also critical to designing effective control measures. However, to date, such factors remain largely understudied.

Responding to WHO recommendations and integrating advanced diagnostic technologies into control programs will support efforts to manage and potentially eliminate *Brugia* spp. infections. Continued advancement and strategic implementation will be key in reducing the associated burden of disease.

## Figures and Tables

**Table 1:** Hosts and geographic ranges for *Brugia* spp. parasites.

Species	Hosts	Geographic Range	References
*B. pahangi*	Domestic cat, wild felids, non-human primates, Asian palm civet (*Paradoxurus hermaphroditus*)	India, Southeast Asia	[15,36,37]
*B. malayi*	Human, non-human primates, domestic cat, domestic dog, Asian palm civet (*Paradoxurus hermaphroditus*), pangolin (*Manis javanica*)	India, Southeast Asia	[10,36,38]
*B. ceylonensis*	Domestic dog	India, Sri Lanka	[16,17]
*B. patei*	Domestic cat, domestic dog, large-spotted genet (*Genetta tigrina*), brown greater galago (*Galago crassicaudatus*)	Kenya (Pate Island)	[24]
*B. beaveri*	Raccoon (*Procyon lotor*), bobcat (*Lynx rufus*), mink (*Neogale vison*)	United States (Louisiana to Florida)	[29]
*B. lepori*	Cottontail rabbits (*Sylvilagus* spp.)	United States (Louisiana)	[30]
*B. tupaiae*	Treeshrews (*Tupaia* spp.)	Southeast Asia	[21]
*B. guyanensis*	South American coati (*Nasua nasua*), greater grison (*Galictis vittata*)	Guyana	[33]
*B. timori*	Human	Lesser Sunda Islands, Indonesia	[39]
*B. buckleyi*	Indian hare (*Lepus nigricollis*)	Sri Lanka	[18]

**Table 2:** Diagnostic features of *Brugia* spp. microfilariae (with two *Wuchereria* spp.).

Species	Length (µm)	Width (µm)	Head	Tail	Acid Phosphatase	References
*B. pahangi*	246–280	5–6	blunt, rounded	tapered	diffuse	[36,37]
*B. malayi*	177–230	5–6	blunt	2 nuclei in tip	excretory pore, anal pore (sometimes amphids, phasmids)	[36]
*B. ceylonensis*	220–275	NR	NR	NR	NR	[17]
*B. patei*	similar to *B. malayi*	NR	NR	2 nuclei in tip	cephalic vesicle, excretory pore, tail	[24]
*B. beaveri*	285–325	4.5–6.5	blunt	NR	NR	[29]
*B. lepori*	275–330	5–7	blunt	2 nuclei in tip	NR	[30]
*B. tupaiae*	283–322	6	blunt	tapered	NR	[21]
*B. guyanensis*	213–232	4–5	blunt	2 nuclei in tip	NR	[33]
*B. timori*	341	6–8	blunt, rounded	tapered	NR	[39]
*B. buckleyi*	NR	NR	NR	NR	NR	[18]
*W. bancrofti*	244–296	6–7	blunt, rounded	tapered	excretory pore, innenkorper, anal pore	[86,87]
*W. kalimantani*	155–208	4–6	rounded	NR	NR	[88]

NR: Not reported.
